# Adoptive Natural Killer Cell Immunotherapy for Canine Osteosarcoma

**DOI:** 10.3389/fvets.2021.672361

**Published:** 2021-06-07

**Authors:** William C. Kisseberth, Dean A. Lee

**Affiliations:** ^1^Department of Veterinary Clinical Sciences, The Ohio State University, Columbus, OH, United States; ^2^Department of Pediatrics, Nationwide Children's Hospital and The Ohio State University Comprehensive Cancer Center, Columbus, OH, United States

**Keywords:** canine, immunotherapy, osteosarcoma, NK cell, TGFβ

## Abstract

Osteosarcoma is the most common primary bone tumor in both humans and dogs. It is a highly metastatic cancer and therapy has not improved significantly since the inclusion of adjuvant chemotherapy into disease treatment strategies. Osteosarcoma is an immunogenic tumor, and thus development of immunotherapies for its treatment, especially treatment of microscopic pulmonary metastases might improve outcomes. NK cells are lymphocytes of the innate immune system and can recognize a variety of stressed cells, including cancer cells, in the absence of major histocompatibility complex (MHC)-restricted receptor ligand interactions. NK cells have a role in controlling tumor progression and metastasis and are important mediators of different therapeutic interventions. The core hypothesis of adoptive natural killer (NK) cell therapy is there exists a natural defect in innate immunity (a combination of cancer-induced reduction in NK cell numbers and immunosuppressive mechanisms resulting in suppressed function) that can be restored by adoptive transfer of NK cells. Here, we review the rationale for adoptive NK cell immunotherapy, NK cell biology, TGFβ and the immunosuppressive microenvironment in osteosarcoma, manufacturing of *ex vivo* expanded NK cells for the dog and provide perspective on the present and future clinical applications of adoptive NK cell immunotherapy in spontaneous osteosarcoma and other cancers in the dog.

## Introduction

Osteosarcoma is the most common primary bone tumor in both humans and dogs, with the disease incidence being as much as 30–50 times higher in the latter (1). The similarities of the disease in humans and dogs are well-described and include commonalities in underlying molecular biology, including gene expression and genetic mutations, histopathology, clinical presentation, disease progression, and response to therapy (2). Spontaneous osteosarcoma in the dog has been used extensively as a preclinical large animal model to evaluate new therapies for osteosarcoma in humans, including limb-sparing procedures (3–5), chemotherapy delivery (6, 7), targeted therapeutics (8), and immunotherapeutics, including therapeutic vaccines and others (9–11). New immunotherapeutic approaches to cancer treatment have emerged and are now making a significant clinical impact for large numbers of cancer patients (12, 13). The development of new immunotherapeutics can be greatly facilitated by the use of well-characterized and validated animal models and spontaneously occurring osteosarcoma in pet dogs is exceptionally well-suited for this purpose. In addition to the similarities described above, other features of the disease in dogs are particularly relevant and important for evaluating immunotherapeutic interventions. Notably, cancers in dogs occur in a relatively outbred population that generally shares similar environmental exposures with humans and the spontaneously occurring tumors are heterogeneous, existing in a complex microenvironment and in a host with an intact immune system; all critical features that are poorly addressed in most rodent models (14).

## Rationale for Adoptive NK Cell Immunotherapy

The core hypothesis of adoptive natural killer (NK) cell immunotherapy is that there exists a natural defect in innate immunity (a combination of cancer-induced reduction in NK cell numbers and immunosuppressive mechanisms resulting in suppressed function) that can be restored by adoptive transfer of NK cells (15). The immunosuppressive tumor microenvironment suppresses NK cell function (16), and although many drugs and radiation can sensitize tumors for recognition by NK cells, chemotherapy, anesthesia, and radiation therapies can also adversely affect NK cell numbers and function (17–21). While much effort has gone into T-cell based approaches for immunotherapy, including chimeric antigen receptor (CAR) T-cells and immune checkpoint inhibition, these approaches can have significant problems that may impede their application such as graft-vs. host disease (GVHD), cytokine release syndrome (CRS), immune effector cell-associated neurotoxicity syndrome (ICANS), or severe on-target off-tissue toxicities (22, 23). Adoptive NK cell therapy is not associated with GVHD (24), thus making it potentially safer than T-cell based therapies and because allogeneic transfer is tolerated, NK cell products can be manufactured and stored for later use in patients as needed, rather than manufacturing “on-demand” for patient-specific use.

## NK Cell Biology

NK cells are lymphocytes of the innate immune system. NK cells can recognize a variety of stressed cells in the absence of major histocompatibility complex (MHC)-restricted receptor ligand interactions. NK cells are non-T, non-B lymphocytes, and are known for their cytotoxicity and cytokine effector functions. Importantly, they can kill target cells without prior antigen sensitization. Also, NK cells can cross-talk with dendritic cells in different ways, thus participating in the shaping of the subsequent immune response. NK cells have a role in controlling tumor progression and metastasis and are important mediators of different therapeutic interventions, including cytokines, antibodies, immunomodulatory drugs, and stem cell transplantation.

### NK Cell Receptors and Function

The number of NK cells as a percentage of peripheral lymphocytes varies widely in humans (1–32.6%, median 7.6%) and in dogs (2.5–15%) (25–29). In humans, NK cells are identified by the lack of CD3 and the presence of CD56 and/or CD16, and make up 85% of the large granular lymphocyte (LGL) population (30). The phenotypic characteristics of NK cells in the dog are not as clearly defined; however, distinct phenotypic NK cell subsets have been described (31). NK cells express many different cell surface receptors that can be grouped as activating, inhibitory, adhesion, cytokine, or chemotactic receptors. Although many of the cell surface molecules involved in the regulation of NK cell function are found in both humans and mice, only a small subset has been validated in the dog. Canine NK cells do express at the mRNA level several genes classically associated with NK cells, such as NKp30, NKp44, NKp46, NKG2D, CD16, DNAM-1, perforin, and granzyme B (25).

The regulation of NK cell function relies on a complex interplay of activating and inhibitory signals. Unlike T-cells, whose activation is highly restricted to an antigenic peptide presented in the groove of MHC proteins, NK cell activation is not antigen specific. NK cell activation and tolerance are accomplished through a large variety of activating receptors for recognition of danger, balanced with an equally large number of inhibitory receptors that identify self. The balance between these signals determines whether NK cells will activate their effector function (e.g., FasL/TRAIL-mediated killing, perforin/granzyme release, or cytokine production). In humans, there are several families of activating receptors, including CD126 (FcRγIIIa), natural cytotoxicity receptors (NCRs), NK Group 2 (NKG2) lectin-like receptors, DNAM-1, and 2B4; however, most of these have not been well-characterized in the dog (32). Activating receptors generally recognize proteins that are upregulated by cell stress or are of non-self-origin, whereas inhibitory receptors primarily bind MHC for self-recognition (33). Inhibitory receptors provide control for NK cell activity against healthy tissue. The primary inhibitory receptors in human NK cells are killer-cell immunoglobulin-like receptors (KIRs) and NKG2A, both of which bind to HLA class I molecules, preventing NK cell-mediated lysis of cells with normal HLA expression (33). MHC class-I deficient targets have heightened sensitivity to NK cell killing. This biology is reflected and summarized by the “missing self” hypothesis, which states that the presence of MHC class I, ubiquitously expressed by healthy cells, provides NK cells with a “self” signal that is recognized by NK cell inhibitory receptors and thus prevents NK cell self-reactivity (34).

### Canine NK Cells

While human NK cells are distinguished by the absence of surface expression of CD3 and the presence of variable levels of expression of CD56 and CD16, depending on differentiation state (35), the phenotypic characterization of canine NK cells is still evolving. Morphologically, canine NK cells are medium- to large-sized lymphocytes containing electron-dense intracytoplasmic granules that contain granzyme B and perforin and lack expression of CD4 and CD20, T-cell and B-cell markers, respectively (36). However, CD8 may be expressed by a subset of these cells (37, 38). Canine NK cell populations have also been defined based on density of CD5 surface marker expression, with CD5^dim^ representing a NK cell population (28), especially in the setting of IL-2 stimulation. Further, under *ex vivo* expansion conditions with cytokine stimulation, the majority of cytotoxic large granular lymphocytes expressed a CD5^dim^CD3^+^CD8^+^TCRαβ^−^TCRγδ^−^CD4^−^CD21^−^CD11c^+/−^CD11d^+/−^CD44^+^ phenotype that highly upregulated NKp46, and expressed traditional T-cell lineage markers, but lacked T-cell receptors (39). NCR1/NKp46, a NK-specific activating molecule, is considered a “pan-species” NK cell marker (40). One study concluded that canine NK cells are comprised of both CD3^−^GranzymeB^+^NCR1^+^ and CD3^−^GranzymeB^+^NCR1^−^ populations cells, with the presence of NCR1/NKp46 positive cells representing an activated state (27, 31). Similarly, a canine-specific antibody to NKp46 identifies CD3^−^NKp46^+^ and CD3^−^NKp46^−^ NK subsets that vary in cytotoxicity, with CD3^−^NKp46^−^ population being less cytotoxic, but could be induced to express NKp46 (25). Another putative marker for canine NK cells is the C-type lectin-like CD94 (KLRD-1). Experiments with a canine-specific anti-CD94 identifies a candidate NK cell population representing ~7.7% of PBMCs and subsets within the CD5^dim^ population (26). It should be noted that the KIR family of surface receptors described above for humans has not been identified in dogs. One gene of a similar paralogous family found in mice, the Ly49 family, has been identified in the canine genome (39, 41).

## Mechanisms of NK Cell Killing

NK cells exert direct and indirect antitumor activity and kill target tumor cells via release of granules containing perforin and granzyme, secretion of cytokines such as IFNγ and other effector molecules, ligation and activation of death receptors, and antibody-dependent cellular toxicity (ADCC) mediated through CD16 when combined with anti-tumor antibodies. Further, the release of pro-inflammatory cytokines enhances the recruitment and maturation of adaptive immune responses (42, 43). The mechanism by which NK cells induce apoptosis in osteosarcoma cells may depend on both the activation state of the NK cells and the death receptor and apoptotic pathways present and functional in the target cell (33). For example, *in vitro*, direct NK cell lysis of osteosarcoma cells is mediated via direct release of granzyme B (44); however, granule-independent mechanisms may be more relevant *in vivo*, as losing Fas and TRAIL may be simpler mechanisms of escape than redundant downstream death pathways (45). Degranulation of NK cells is mediated by the balance of activating and inhibitory receptors, which in turn is influenced by the expression of ligands on the tumor cell. This suggests that NK cells isolated, expanded, and activated using different techniques may differ as to which activating receptors are highly expressed and important for recognizing a particular tumor (33). For example, in one study IL-15 stimulated NK cells targeted osteosarcoma predominantly through DNAM1, whereas in another study IL-2 stimulated NK cells targeted osteosarcoma predominantly through NKG2D (44, 46).

## Osteosarcoma, the Immunosuppressive Microenvironment, and TGFβ

Tumors, especially solid tumors, have evolved mechanisms to actively suppress the immune system. These include induction of inhibitory receptors on NK and T-cells, recruitment of Tregs, myeloid derived suppressor cells and tumor associated macrophage, and production of immunosuppressive cytokines and other factors, including TGFβ (47). Overexpression of TGFβ is a hallmark of many cancers, including osteosarcoma. It inhibits NK cell activity through several mechanisms-suppressing NKG2D and CD16 expression, decreasing perforin, and inhibiting cytokine release (48–51). TGFβ is highly expressed in cancer cell lines and notably, is more highly expressed in osteosarcoma than most other solid tumor cell lines, suggesting that TGFβ is an important contributor to the immunosuppressive tumor microenvironment for osteosarcoma in particular (52). TGFβ signaling is a crucial factor in cross-talk between osteosarcoma cells and stroma cells. Secretion of TGFβ by tumor cells or stroma cells can act in a paracrine manner to regulate the tumor microenvironment, promoting angiogenesis, bone remodeling and cell migration, and by inhibiting immunosurveillance. TGFβ secretion and TGFβ receptor expression has been demonstrated in canine osteosarcoma cells (53). Our group has developed a NK cell expansion technique that confers relative TGFβ-resistance to NK cells in an attempt to improve their function in the hostile immunosuppressive tumor microenvironment (54). TGFβ resistance, or imprinting, is achieved by chronic exposure of NK cells to IL-2 and TGFβ during the expansion process. TGFβ-imprinted NK cells secrete more IFNγ and TNFα than non-imprinted NK cells in the absence, or presence, of TGFβ. Furthermore, TGFβ-imprinted NK cells have increased cellular toxicity compared to non-imprinted cells and are more resistant to TGFβ-mediated decreases in cellular cytotoxicity (54). *Ex vivo* expanded canine NK cells cultured under similar conditions are likewise conferred relative TGFβ-resistance (Lee, unpublished).

## Manufacturing of *Ex vivo*-Expanded Canine NK Cells for Adoptive Immunotherapy

NK cells for clinical use can be obtained through apheresis with T-cell depletion, or by *ex vivo* expansion. In humans, NK cells have been successfully expanded from peripheral blood, cord blood, and pluripotent or embryonic stem cells (55). Various methods for expanding purified NK cell populations have been developed in people, using exposure to different cytokines and co-culture with feeder cell lines (15, 55). Several of these methods have been extrapolated to and modified for canine studies (25, 56). In general, superior expansion is achieved when recombinant canine cytokines are used vs. recombinant human cytokines. In humans, the incorporation of IL-21 cytokine exposure by co-culture with the K562 feeder cell line, significantly enhanced NK cell expansion in IL-15/IL-2 expanded NK cells (55).

**Figure 1:**
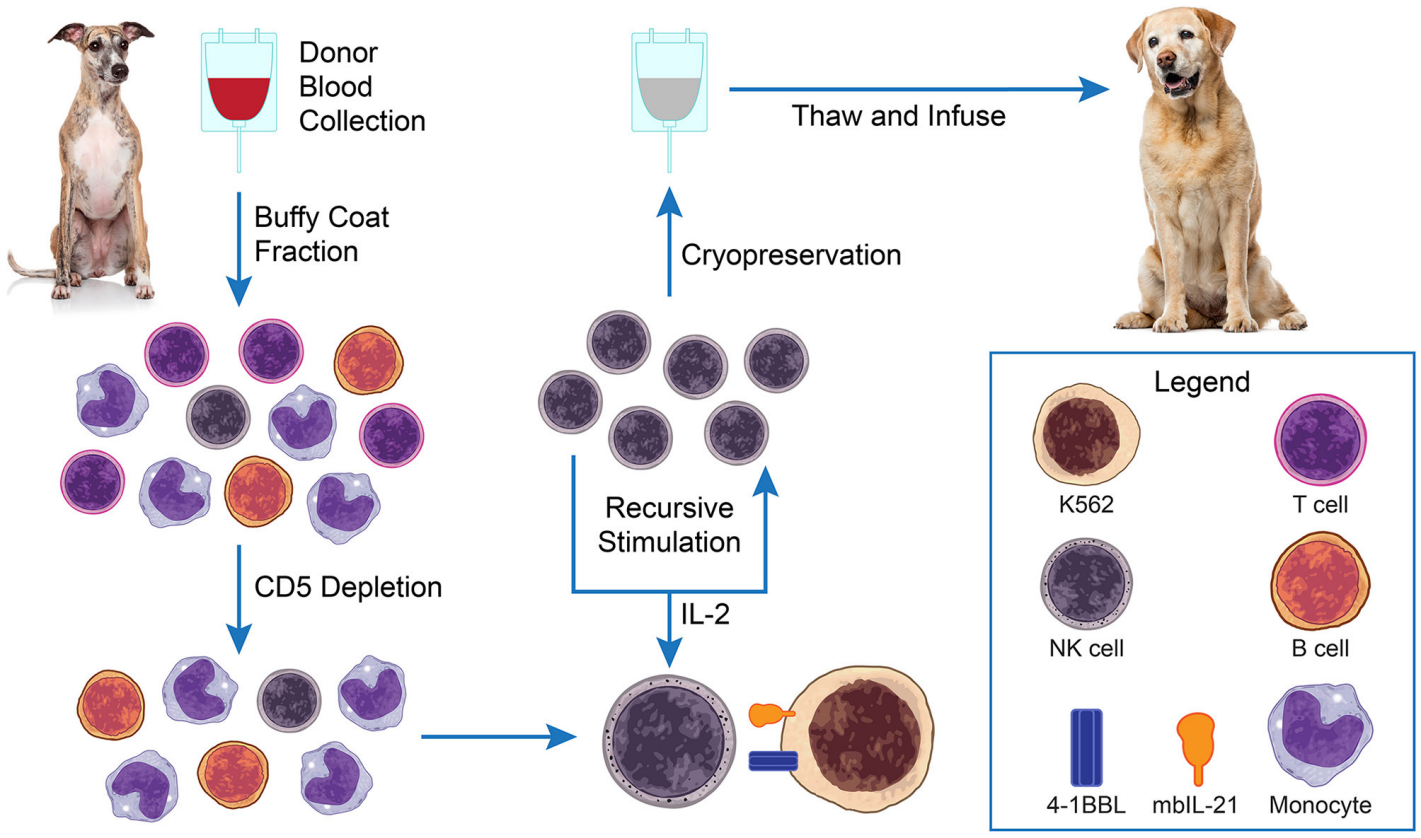
Adoptive natural killer (NK) cell therapy. PBMCs are isolated from blood buffy coats of healthy blood donor dogs by Ficoll separation. The separated cells undergo CD5 cell depletion and are then co-cultured and recursively stimulated in the presence of IL-2 (±TGFβ) 3× over a 3-week period with irradiated K562 feeder cells expressing membrane bound IL-21. Expanded NK cells can be used immediately, or cryopreserved for later use.

These techniques have been modified by our group to manufacture *ex vivo* expanded canine NK cells with similarly robust results (25). Clinical grade expanded NK cells are produced using good manufacturing practices (GMP) principles including closed-system processes and standardized release testing and certification criteria. For our studies, the primary donor NK cells used for expansion are obtained from the buffy coats of routine whole blood donations from healthy volunteer canine blood bank donors at our veterinary medical center. Peripheral blood mononuclear cells (PBMCs) are isolated by Ficoll separation from the buffy coats. The separated cells undergo CD5 cell depletion and are then co-cultured and recursively stimulated in the presence of IL-2 three times over a 3 week period with irradiated human K562 feeder cells expressing the co-stimulatory ligand 4-1BBL and membrane bound IL-21 (Figure 1). The final *ex vivo* expanded NK cell product release criteria include: ≥70% viability, CD3+ cells <5%, NKp46+ cells as reported, endotoxin <5 EU/kg, and mycoplasma negative. Expanded NK cells can be used immediately, or cryopreserved for later use.

The influence and importance of the donor on the final NK cell product is largely unexplored, although there does appear to be individual donor variability in the robustness of expansion and *in vitro* cytotoxicity of the final expanded NK cell product. The influence of donor breed is also unknown. A preliminary survey of NK cell numbers (NKp46+, CD3–) and expression of DNAM-1 and TIM-3 receptors in the four most common donor breeds (greyhound, pit bull, golden retriever, and Labrador retriever) in our blood bank showed minor breed differences with no one breed being a clearly superior donor source (Peck, unpublished). Ultimately, for our initial clinical trials, we chose to use greyhound donors exclusively, as they are the most common breed in our blood donor population and by doing so, any unknown breed associated variability in the final product could be excluded.

## Clinical Application of Adoptive NK Cell Immunotherapy in Canine Osteosarcoma

As described above, spontaneous osteosarcoma in pet dogs provides an ideal large animal translational model for studying new immuno-oncology approaches for treating this cancer, including adoptive NK cell therapy. The more recent development of canine-specific antibodies for identifying canine NK cells and subsets, adaptation and development of *ex vivo* NK cell expansion techniques, and overall gradually increasing availability of canine-specific reagents and analysis techniques, now makes clinical trials of adoptive NK cell therapy for osteosarcoma and other cancers in dogs more feasible and informative. However, these studies and trials are only just now beginning.

The first reported clinical trial of adoptive NK cell immunotherapy in dogs with appendicular osteosarcoma evaluated autologous *ex vivo* expanded NK cells administered intra-tumorally following completion of a hypo-fractionated palliative radiation protocol (56). In this study, NK cells were isolated and expanded from the canine patient using an expansion technique similar to that described above (56). Two injections of 7.5 × 10^6^ NK cells/kg were co-injected with 250,000 IU/kg of rhIL-2. Ten dogs were treated in this study. Overall, there was limited systemic toxicity with this protocol. One dog had a grade 3 reaction of fever, chills, excessive salivation, and dehydration consistent with IL-2 toxicity. Three dogs had local infection/tissue breakdown at the NK cell injection site. Persistence of labeled viable NK cells could be demonstrated in tumor biopsies performed 1 week after intra-tumoral injection. Interestingly, analysis of PBMCs pre- and post-treatment demonstrated a significant increase in circulating granzyme B+ CD45+ cells (56).

Our group recently opened a phase I clinical trial of intravenously administered allogeneic TGFβ-resistant (imprinted) NK cells combined with adjuvant carboplatin chemotherapy in dogs with appendicular osteosarcoma receiving limb amputation (54). In this trial, dogs receive a single dose of NK cells 24 h prior to amputation to evaluate NK cell trafficking to the primary tumor. Three additional doses are administered during the subsequent 48 h post-amputation period. Dogs then receive standard adjuvant carboplatin chemotherapy every 3 weeks, with additional NK cell doses administered on the weeks they are not receiving chemotherapy. In total, dogs receive a total of twelve doses of adoptive NK cells—significantly more doses than in most human NK cell immunotherapy trials, to date. The use of allogeneic NK cells greatly increases the yield and potential cell doses, reduces the cost of therapy, and simplifies the logistics for delivery. Although this approach requires cryopreservation of the product which may impact NK cell viability and function, we have successfully used cryopreserved NK cells for several human studies (57, 58).

One of the major strengths of clinical trials in dogs with spontaneously occurring cancers is the ability to do intensive longitudinal patient biospecimen sampling and clinical assessments, often more intensively than is possible in a comparable clinical trial in human patients. This is well-illustrated in the afore described first-in-dog clinical trial, where pre- and post-treatment serum cytokine concentrations were assessed by ELISA, tumor gene expression profiles by qRT-PCR, circulating immune cell phenotypes by flow cytometry, and intra-tumoral immune cell phenotypes by immunohistochemistry and qRT-PCR (59). Gradually increasing availability of new canine-specific reagents and application of new technologies to the dog, will further increase the number and power of the correlative studies that can be done and their translational relevance.

Understanding of the pharmacokinetics and trafficking of adoptively transferred NK cells and consequent effects on systemic and tumor immune cell phenotypes and responses to therapy are important biological correlates for assessing adoptive NK cell strategies and in principle can be addressed in this model using a variety of approaches. Assessment of circulating NK cell numbers and phenotypes in blood can be assessed by flow cytometry; however, distinguishing donor from patient cells is problematic. Optimization of variable number tandem repeat PCR assays as is used to assess tissue chimerism in human transplant patients (60) and experimental canine bone marrow transplant models (61–63), may be useful for assessing the relative circulating donor NK cell component. Sex chromosome (XX/XY) FISH chimerism testing may be another method that could be applied when there is a sex-mismatched donor (64). Novel cell labeling agents have been developed and tested in rodent and non-human primate models and could be useful for evaluating NK cell kinetics and trafficking in the canine osteosarcoma model. *Ex vivo*-expanded human NK cells labeled with the non-radioactive isotope fluorine 19 (^19^F) can be detected in rodent tissues by NMR and imaged with ^19^F-MRI (65, 66). Similarly, expanded NK cells from rhesus macques were labeled with ^89^zirconium-oxine (^89^Zr-oxine) cell labeling and quantitated and imaged with positron emission tomography (PET)/CT (67).

## Future Applications

While these early studies of adoptive NK cell therapy in dogs are demonstrating the feasibility, tolerability, and safety of the approach, the model is well-suited for investigating many ongoing and new important questions in the field. As one of the mechanisms by which NK cells kill cancer cells is ADCC, combining adoptive NK cell therapy with therapeutic antibodies is of interest. Studying ADCC in spontaneous canine cancers may be feasible in some cases with murine, chimeric, or humanized antibodies, as canine Fc gamma receptors bind to dog, human, and mouse IgGs. However, caninized therapeutic antibodies may be preferred, as species differences in affinity may result in significant differences in activity, and eventual alloimmunization and neutralization by the host may significantly alter the antibody half-life of non-canine antibodies (68, 69). Of great interest is the genetic modification of NK cells to express chimeric antigen receptors (CAR) to target and enhance their killing (70, 71). Clinical trials investigating this approach are in their early stages in people. However, investigation of new CAR-NK constructs in dogs with osteosarcoma could address questions of toxicity, tumor targeting, immunologic response and anti-tumor activity. An important hurdle to genetic modification of NK cells has been their relative resistance to lentiviral and retroviral transduction (24, 72). Our group recently developed a method for genome editing of human primary and expanded NK cells using Cas9 ribonucleoprotein complexes (Cas9/RNPs) that allows for efficient knockout of genes in NK cells, thus opening the door for novel and innovative genetic modification strategies, including modifications that would affect tumor targeting and NK cell activation state, *in vivo* proliferative capacity, and cytotoxicity (73, 74). As the use of genetically modified cells in humans has even more significant regulatory hurdles to overcome compared to similar trials in dogs, clinical trials in dogs can speed the evaluation of novel approaches, identify those that are more promising, and provide additional useful safety data to inform subsequent human trials. As noted above, because of the ability to easily acquire patient biospecimens, including tumor biopsies, and the relatively comparable size to humans, the model is ideal for investigating effects on tumor targeting achieved with different CAR-NK constructs and for studying novel NK cell labeling and imaging techniques (65–67).

## Data Availability Statement

The original contributions presented in the study are included in the article/supplementary material, further inquiries can be directed to the corresponding author/s.

## Author Contributions

WK and DL contributed equally to the conception of this perspective and wrote sections of the manuscript. WK wrote the first draft of the manuscript. Both authors contributed to manuscript revision, read, and approved the submitted version.

## Conflict of Interest

DL holds stocks and options in Courier Therapeutics, Caribou Biosciences, and Kiadis Pharma; received consulting fees from Kiadis Pharma; received research grants from Kiadis Pharma; and has intellectual property licensed to Kiadis Pharma from which royalties are received. The remaining author declares that the research was conducted in the absence of any commercial or financial relationships that could be construed as a potential conflict of interest.
